# Supplementary figures and images for: A mixed-methods study of factors influencing postpartum intrauterine device uptake after family planning counseling among women in Kigali, Rwanda
Source: PLoS One. 2022 Nov 3;17(11):e0276193. doi: 10.1371/journal.pone.0276193 (PMC9632907; doi:10.1371/journal.pone.0276193)

## Flowchart for case-control study Participant Recruitment


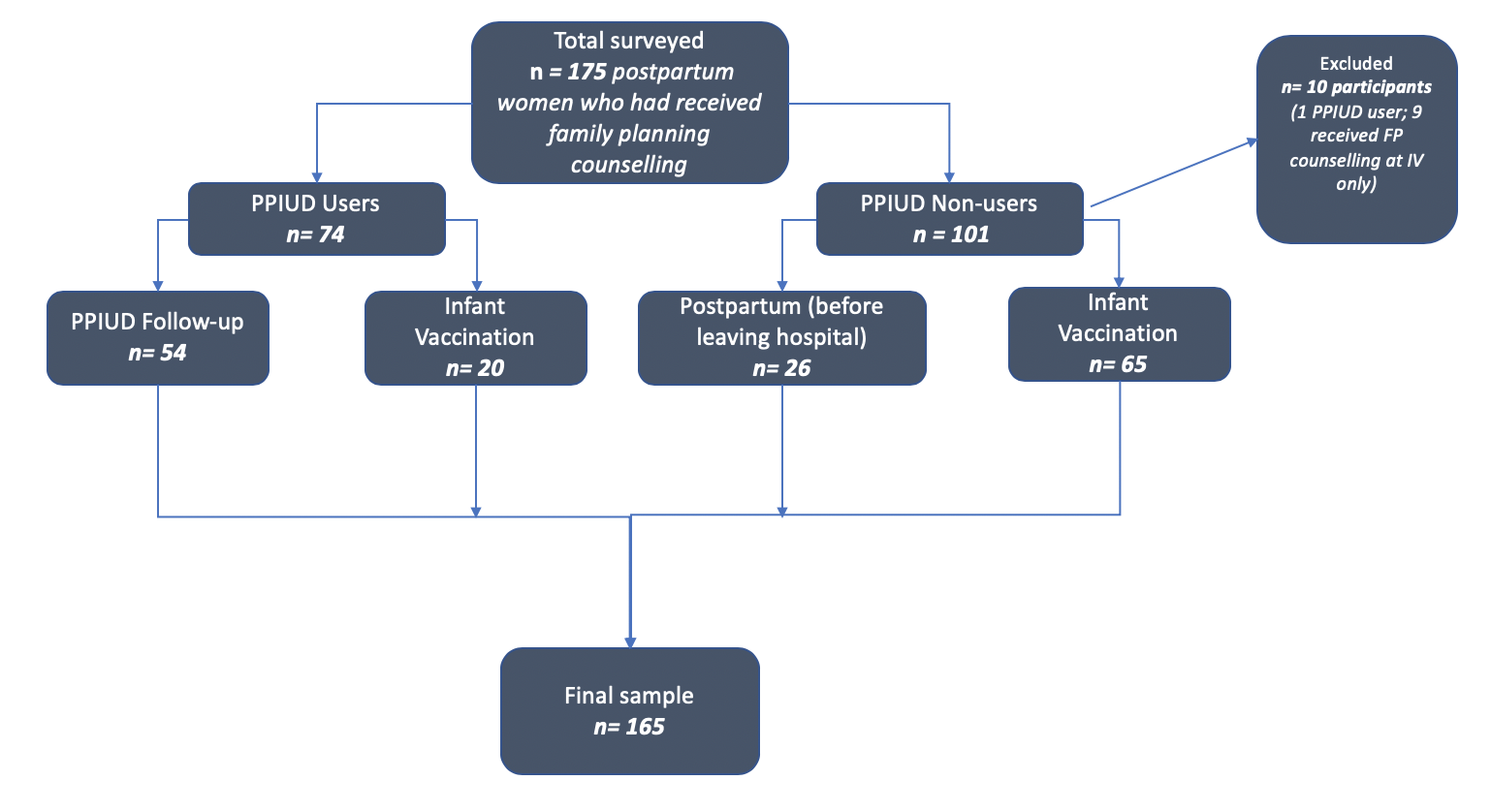

Supplement: S1 Fig — (DOCX) [file pone.0276193.s001.docx]
