# Supplementary material for: A mixed-methods study of factors influencing postpartum intrauterine device uptake after family planning counseling among women in Kigali, Rwanda
Source: PLoS One. 2022 Nov 3;17(11):e0276193. doi: 10.1371/journal.pone.0276193 (PMC9632907; doi:10.1371/journal.pone.0276193)
Supplement: Attachment — Submitted filename: ResponseToReviewers_16Sept22.docx [file pone.0276193.s004.docx]

**1. Please note that funding information should not appear in the Acknowledgments section/Funding section or Any other areas of your Manuscript. We will only publish funding information present in the Funding Statement section of the online submission form. Please remove any funding-related text from the manuscript.**

We have removed funding information from the manuscript. **2. Please include your tables as part of your main manuscript and remove the individual files. Please note that supplementary tables (should remain/ be uploaded) as separate "supporting information" files**

The tables are now included as part of the main file. **3. Please include the following request in the decision letter, and ping me (lverduci@plos.org) with follow up. “Please include a complete copy of PLOS’ questionnaire on inclusivity in global research in your revised manuscript. Our policy for research in this area aims to improve transparency in the reporting of research performed outside of researchers’ own country or community. The policy applies to researchers who have travelled to a different country to conduct research, research with Indigenous populations or their lands, and research on cultural artefacts. The questionnaire can also be requested at the journal’s discretion for any other submissions, even if these conditions are not met. Please find more information on the policy and a link to download a blank copy of the questionnaire here:** [**https://nam11.safelinks.protection.outlook.com/?url=https%3A%2F%2Fjournals.plos.org%2Fplosone%2Fs%2Fbest-practices-in-research-reporting&amp;data=05%7C01%7Ckmwall%40emory.edu%7C52cb0b6d24e54e42771008da96c5cffd%7Ce004fb9cb0a4424fbcd0322606d5df38%7C0%7C0%7C637988073594233277%7CUnknown%7CTWFpbGZsb3d8eyJWIjoiMC4wLjAwMDAiLCJQIjoiV2luMzIiLCJBTiI6Ik1haWwiLCJXVCI6Mn0%3D%7C3000%7C%7C%7C&amp;sdata=CceucGxcuYFBnMzC4qyBSY30bMBb3mys%2BgIagr2A60s%3D&amp;reserved=0**](https://nam11.safelinks.protection.outlook.com/?url=https%3A%2F%2Fjournals.plos.org%2Fplosone%2Fs%2Fbest-practices-in-research-reporting&amp;data=05%7C01%7Ckmwall%40emory.edu%7C52cb0b6d24e54e42771008da96c5cffd%7Ce004fb9cb0a4424fbcd0322606d5df38%7C0%7C0%7C637988073594233277%7CUnknown%7CTWFpbGZsb3d8eyJWIjoiMC4wLjAwMDAiLCJQIjoiV2luMzIiLCJBTiI6Ik1haWwiLCJXVCI6Mn0%3D%7C3000%7C%7C%7C&amp;sdata=CceucGxcuYFBnMzC4qyBSY30bMBb3mys%2BgIagr2A60s%3D&amp;reserved=0)**. Please upload a completed version of your questionnaire as Supporting Information when you resubmit your
manuscript.**

We have uploaded a completed version of this questionnaire as Supporting Information.
